# Immunoadsorption therapy in patients with multiple sclerosis with steroid-refractory optical neuritis

**DOI:** 10.1186/1742-2094-9-80

**Published:** 2012-04-26

**Authors:** Michael J Koziolek, Desiree Tampe, Matthias Bähr, Hassan Dihazi, Klaus Jung, Dirk Fitzner, Reinhard Klingel, Gerhard A Müller, Bernd Kitze

**Affiliations:** 1 Department of Nephrology and Rheumatology, Georg-August-University Göttingen, Robert-Koch-Strasse 40, D-37075, Göttingen, Germany; 2 Department of Neurology, Georg-August-University Göttingen, Robert-Koch-Strasse 40, D-37075, Göttingen, Germany; 3 Department of Medical Statistics, Georg-August-University Göttingen, Robert-Koch-Strasse 40, D-37075, Göttingen, Germany; 4 Apheresis Research Institute, Stadtwaldguertel 77, 50935, Cologne, Germany

**Keywords:** Apheresis, Autoimmune diseases, Evoked potentials/visual, Immunoadsorption, Multiple sclerosis, Optic neuritis, Proteomics

## Abstract

****Background**:**

In multiple sclerosis relapses refractory to intravenous corticosteroid therapy, plasma exchange is recommended. Immunoadsorption (IA) is regarded as an alternative therapy, but its efficacy and putative mechanism of action still needs to be established.

****Methods**:**

We prospectively treated 11 patients with multiple sclerosis who had optical neuritis and fulfilled the indications for apheresis therapy (Trial registration DE/CA25/00007080-00). In total, five IA treatments were performed using tryptophan-IA. Clinical activity (visual acuity, Expanded Disability Status Scale, Incapacity Status Scale), laboratory values and visual evoked potentials were measured before, during and after IA, with a follow-up of six months. Moreover, proteomic analyses were performed to analyze column-bound proteins as well as corresponding changes in patients’ sera.

****Results**:**

After the third IA, we detected an improvement of vision in eight of eleven patients, whom we termed responders. Amongst these, the mean visual acuity improved from 0.15 ± 0.12 at baseline to 0.47 ± 0.32 after the third IA (*P* = 0.0252) up to 0.89 ± 0.15 (*P* < 0.0001) at day 180 ± 10 after IA. Soluble interleukin-2 receptor decreased in responders (*P* = 0.03), whereas in non-responders it did not. Proteomic analyses of proteins adsorbed to IA columns revealed that several significant immunological proteins as well as central nervous system protein fragments, including myelin basic protein, had been removed by IA.

****Conclusions**:**

IA was effective in the treatment of corticosteroid-refractory optic neuritis. IA influenced the humoral immune response. Strikingly, however, we found strong evidence that demyelination products and immunological mediators were also cleared from plasma by IA.

## **Background**

Previous studies have described four immunopathological patterns of demyelination in early multiple sclerosis (MS) lesions, with pattern II being characterized by antibody and/or complement-associated demyelination [1]. Several specific antibodies have been described and discussed to contribute to the humoral autoimmune response in MS [2]. Immunoglobulins are synthesized intrathecally; however, at least part of the humoral response in MS is derived systemically from the blood [3].

Therapeutic plasma exchange (PE) is based on the separation of plasma from cellular blood components, allowing the removal of substances up to a molecular weight of 3 × 10^3^ kDa. As shown in a randomized placebo controlled cross-over study, PE was efficient for steroid - refractory relapses in about 40% to 50% of cases of acute central nervous system inflammatory demyelinating diseases [4]. The usefulness of PE has also been extended to severe optic neuritis in patients with MS [5,6]. Thus, the use of PE in steroid-refractory relapses has become an integral part of European guidelines for the treatment of MS [7]. Clinical-pathological correlation analyses have shown that all patients with pattern II pathology but none with pattern I or pattern III experienced improvement in neurological deficits after being treated with PE [8]. This selective response suggests a removal of pathogenic humoral and plasma factors by PE.

Immunoadsorption (IA) provides a more selective approach and the potential for technical innovations in therapeutic apheresis techniques, allowing the elimination of pathogenic antibodies while sparing other plasma proteins. With IA, relevant side effects of PE resulting from protein substitution can be avoided [9]. We hypothesized that IA is at least equally efficient compared to PE as an escalation therapy for steroid-unresponsive relapses of MS. Therefore, we performed a prospective trial to compare IA treatment in 11 patients with MS with our earlier patient population treated with PE for their MS [10]. In addition, proteomic analyses of column-bound proteins were performed as well as measurements of corresponding changes in patients’ plasma samples.

## **Methods**

### **Patients**

We prospectively included 11 consecutive patients with MS who had functionally disabling acute optical neuritis. Patients fulfilled the indications for apheresis treatment due to this steroid-unresponsive MS relapse according to German guidelines (http://www.dgn.org) [7].

The study protocol had been approved by the local ethics committee prior to study initiation (no. 2/4/07) and registered at the local government (no. DE/CA25/00007080-00). All patients gave their written informed consent before enrolment.

### **Immunoadsorption treatment**

IA was performed using the tryptophan-linked polyvinyl alcohol adsorber TR-350, after membrane plasma separation with the polyethylene plasma separator OP-05 W (Asahi Kasei Kuraray, Tokyo, Japan) in combination with the Octo Nova extracorporeal circuit technology (SW 4.30.2, front 4.30.0) (Diamed Medizintechnik, Cologne, Germany). The adsorber, plasma separator and tubing system were for single use only. Combined anticoagulation, with citrate and unfractionated heparin, was used for all treatments. The treated plasma volume was 2,500 mL plasma for all treatments of all patients. In total, five sessions were performed in each patient on alternate days. In case of complications or decrease of fibrinogen below 100 mg/dL, treatment-free intervals were extended individually. Internal jugular veins were used for central vascular access with double lumen catheters in all patients.

### **Baseline and follow-up visits**

All patients were followed up by a neurologist. Visits were performed at baseline (visit 0), after each IA treatment (visits 2 to 5) and after 30 ± 5 days (visit 6), 60 ± 10 days (visit 7) and 180 ± 10 days (visit 8). The neurological findings were assessed using the Expanded Disability Status Scale (EDSS) and the Incapacity Status Scale (ISS) [11-13]. Changes of visual acuity were monitored using standardized near vision types after correction of refractive error at each visit and confirmed by an ophthalmologist before and after IA, as well as at 180 ± 10 days. Visual evoked potentials were determined at baseline, after the last IA and 60 ± 10 days post-intervention with the Neuropack M1 (Nihon Koden, Surbiton, UK).

### **Classification of side effects**

Side effects were defined as any unexpected or symptomatic event that had a possible, probable or definite causal relationship with IA treatment [14]. They were classified as mild, moderate or severe as described previously [15] with small modifications. Briefly, mild side effects included those of transient nature with little or no clinical significance and without any temporary break of the procedure. Side effects that required medical intervention but were not life-threatening were classified as moderate. Unstable and life-threatening events requiring termination of the procedure were classified as severe.

### **Clinical chemistry**

All laboratory parameters were measured by standard methods. The complement components C3c and C4 as well as the immunoglobulins G (IgG), A (IgA) and M (IgM) were measured by nephelometry (Behring Nephelometer II Analyzer, Germany). Soluble interleukin-2 receptor (sIL-2R) was detected on an immulite system (Siemens, Germany).

### **Identification of immunoadsorption column-binding proteins by elution and proteomics**

Immusorba TR-350 column-binding proteins were eluted after the first IA treatment in five of eleven patients. Prior to elution, the column was washed with PBS buffer. The protein elution was carried out as following: PBS-washing step was followed by a three-step elution protocol using solution A (100 mM sodium acetate, 1 M NaCl, pH 5), solution B (20 mM Tris–HCl, 1 M NaCl, pH 8.5) and solution C (20% acetonitril in double diluted H2O). Proteins eluted from all three steps were pooled together and aliquots of 10 mL were used for protein precipitation, protein estimation and two-dimensional gel electrophoresis.

Two-dimensional gel electrophoresis, protein visualization and image analysis, in-gel digestion, mass spectrometry analysis of the digestion products and protein identification using a database search were performed as described in detail in our previous publication [16]. For protein identification, qualitative criteria encompassed optimized mass accuracy (<50 ppm), minimal mass deviation (in the millidalton range) and highest possible probability score, which were assigned to each identified protein. Proteins identified by mass spectrometry are described qualitatively.

### **Western blot analysis**

Western blot analyses were performed according to previously published data [17] with 20 μg of plasma proteins. Equal loading was ascertained by Coomassie staining. For immunodetection of proteins, the following antibodies were used: rabbit polyclonal to human CD5 ligand (CD5L) and mouse monoclonal to human myelin basic protein (both Abcam, Cambridge, UK); and horseradish peroxidase-linked donkey anti-rabbit antibody (Amersham Biosciences, Freiburg, Germany). Results are expressed as mean ± SD.

### **Statistics**

Visit and response effects were studied by two-way repeated measures analysis of variance using the mixed procedure for the software SAS (version 9.1, SAS Institute). In this global analysis, *P* < 0.05 indicated a significant effect. In the case of a significant effect at a particular visit, subsequent pairwise comparisons to baseline values were performed using one-way repeated measures analysis of variance to detect the point in time when the effect occurred. These comparisons were performed at Bonferroni-adjusted significance levels (related *P*-values are labeled with either Bonf. sig. (significant) or with Bonf. n.s. (non-significant)).

Smoothing splines with three degrees of freedom were fitted to mean values of each visit to illustrate the trends and the potential interactions between visits and response effects. Fitting was performed using the free software R (version 2.8, http://www.r-project.org).

Comparative statistical analyses of changes within-subject and within-treatment were performed using *t*-tests for paired samples in case of normal data and Mann–Whitney *U*-tests for non-normal data. Normality was checked by quantile-quantile plots. Again, test results with *P <* 0.05 were considered significant.

## **Results**

IA treatments were started after a mean time of 26.6 days after the initial symptoms and 10.8 days after the start of corticosteroid therapy. Each patient received at least two courses of corticosteroid therapy prior to IA therapy, with a mean cumulative dose of 10.9 g prednisolone equivalent but without a significant improvement, thus fulfilling the indication of therapeutic apheresis as adjunct treatment. An improvement of visual acuity up to 0.6 or more was achieved in eight of eleven patients (72.7%) undergoing IA treatment (responder group). Two patients did not respond to therapy at any time point and one patient improved during IA therapy but deteriorated shortly after the end of IA therapy, associated with the incidence of a jugular venous thrombosis on the same side (non-responder group). The relevant data of patients’ characteristics are shown in Table 1.

**Table 1:** Clinical baseline characteristics of the participants

**Parameter**	**Total (n = 11)**	**Responder (n = 8)**	**Non-responder (n = 3)**
Gender			
* Female*	7 (64%)	5 (62%)	2 (67%)
* Male*	4 (36%)	3 (38%)	1 (33%)
Age	33.6 ± 10.5	29.6 ± 9.2	44.3 ± 8.0
	(19.0 to 55.0)	(19.0 to 47.0)	(39.0 to 55.0)
Body mass index	25.5 ± 3.0	22.9 ± 2.6	26.2 ± 4.0
	(20.1 to 30.7)	(22.0 to 30.7)	(20.1 to 25.7)
Relapse rates in the past 12 months (median and range)	2	2	1
	(1 to 4)	(1 to 4)	(1 to 2)
Baseline Expanded Disability Status Scale	4	3.5	4
	(2 to 7.5)	(2 to 7.5)	(3 to 4)
Days after onset of relapse	26.6 ± 14.6	29.3 ± 15.6	19.3 ± 10.1
	(10.0 to 60.0)	(10.0 to 60.0)	(10.0 to 30.0)
Days after initiation of corticosteroid therapy	10.8 ± 6.9	12.3 ± 6.6	7.0 ± 7.2
	(1.0 to 20.0)	(3.0 to 20.0)	(1.0 to 15.0)
Cumulative dose of prednisolone equivalents (g)	9.5 ± 3.7	8.6 ± 3.7	12.0 ± 2.6
	(4.0 to 15.0)	(4.0 to 15.0)	(10.0 to 15.0)
Duration of disease (years)	3.5 ± 4.9	5.7 ± 2.9	2.7 ± 9.0
	(0.0 to 16.0)	(0 to 7.0)	(0.0 to 16.0)
Initial disease modifying therapy	3 (27.3%)	2 (25%)	1 (33.3%)
Interferon beta	2 (18.2%)	2 (25%)	0 (0%)
Glatiramer acetate	1 (9.1%)	0 (0%)	1 (33.3%)

Descriptive values are shown either as absolute numbers (frequencies) or mean ± standard deviation (minimum –maximum), except for baseline EDSS and relapse rates which are shown as medians and ranges.

### **Visual acuity**

**Figure 1:**
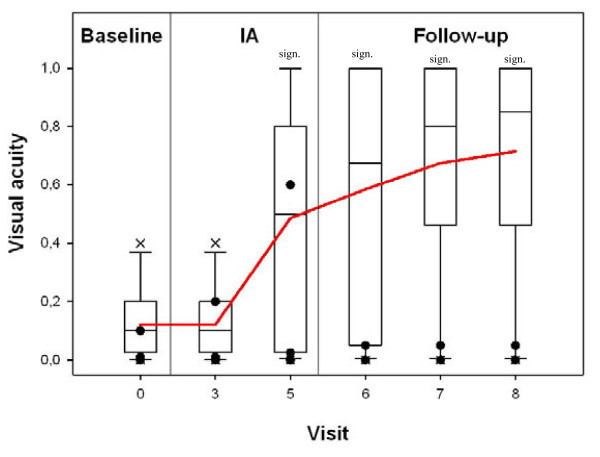
**Time-course of visual acuities of the affected eyes assessed by standard charts in 11 patients with multiple sclerosis treated by immunoadsorption.** Visual acuities were shown as box plots (outliers marked as X) at baseline (visit 0), after three IA therapies (visit 3), after five IA therapies (visit 5), after 30 days (visit 6), after 60 days (visit 7), and after 180 days (visit 8). Three patients did not show long-term improvement of visual acuity, they were termed non-responders in this study (shown as filled circles, partially overlapping each other). AE: affected eyes; sign.: significant (pairwise comparison to baseline).

Mean visual acuity of all patients significantly improved with a baseline value of 0.12 ± 0.12 before the start of IA, 0.36 ± 0.33 (*P* = 0.0234) after the third IA and 0.72 ± 0.38 (day 180 ± 10 after IA; *P* < 0.001). The effect was even more pronounced in the responder group. Mean visual acuity before start of IA was 0.15 ± 0.12 with an increase after the third IA to 0.47 ± 0.32 (*P* = 0.0252 Bonf. n.s.) and even more up to 0.89 ± 0.15 (*P* < 0.0001 Bonf. sig.) at day 180 ± 10 after IA. By contrast, in the non-responder group, no significant changes of visual acuity were detectable. Results are summarized in Figure 1.

Relief of visual impairment inversely correlated with the time interval between the start of symptoms and the beginning of IA therapy (r = -0.539) with a trend (*P* = 0.08) favoring positive clinical response in early opposed to late IA initiation.

### **Visual evoked potentials**

Before IA treatment, visual evoked potentials could not be identified in six of our patients with MS due to the severity of the optical neuritis. However, after treatment in four of these participants, potentials recovered at day 60. In five patients, the visual evoked potential amplitudes as well as latencies before and after IA could be compared. The mean amplitude was 2.31 ± 2.57 mV at baseline, 3.34 ± 2.60 mV after the last IA, and 5.69 ± 1.76 mV at day 60 (*P* = 0.37). Latencies did not show recovery in those five patients from baseline (107.6 ± 21.7 ms) until day 60 ± 10 (112.3 ± 17.1 ms).

### **Expanded disability status scale and incapacity status scale**

The EDSS continuously improved in the responder group starting from 4.06 ± 1.82 at baseline to 3.44 ± 2.38 after the last IA to a minimum of 2.81 ± 2.72 at day 60 ± 10 (*P* = 0.58), in contrast to the non-responder group who showed no changes. However, EDSS is clearly dominated by ambulation [12] and rather insensitive for visual dysfunction [13]. In parallel, ISS ameliorated in the responder group from 8.00 ± 9.02 at baseline with a continuous decrease to a minimum of 5.75 ± 6.78 at day 180 ± 10 (*P* = 0.04), in contrast to no significant changes in the non-responder group.

### **Side effects**

In total, 55 IA treatments were analyzed. Mild side effects were often reported and caused slight discomfort to the patients, such as coldness, cough, dizziness, headache, temporary hypertension, lacrimation, muscular cramps, nausea, palpations, pruritus, paresthesia, rhinorrhea, sweating, tinnitus or vomiting. They were transient and could be easily managed without therapy.

Ten moderate side effects were recorded during the IA phase. These included vascular access problems such as central venous catheter infection and jugular venous thrombosis as well as therapy-associated events that included chest pain, dyspnoe, transient hypotension and urticaria, which could be easily managed by approved medical interventions. Two patients developed febrile infections (common cold and phlebitis of a peripheral vein) within 24 h of IA, which were possibly related to short-term immune dysfunction caused by corticosteroid therapy and IA. No severe side effects occurred. Results are summarized in Table 2.

**Table 2:** Moderate adverse events occurring during immunoadsorption that were classified as being related to vascular access, the immunoadsorption procedure or immunosuppression

	**Patients (n = 11)**	**Immunoadsorption therapies (n = 55)**
**Vascular access-related**		
Central venous catheter exit site infection	1 (9.1%)	1 (1.8%)
Jugular vein thrombosis	1 (9.1%)	1 (1.8%)
**Side effects during immunoadsorption therapy**		
Chest pain	1 (9.1%)	1 (1.8%)
Dyspnoe	1 (9.1%)	1 (1.8%)
Transient hypotension	4 (36.5%)	5 (9.1%)
Urticaria	1 (9.1%)	1 (1.8%)
**Putatively related to immunosuppression both by steroids and immunoadsorption**		
Febrile infection	2 (18.2%)	2 (3.6%)

### **Laboratory data**

In routine analyses, no significant changes were seen for the following values: partial thromboplastin time, hemoglobin, hematocrit, thrombocytes, sodium, calcium, creatinine, aspartate aminotransferase, alanine aminotransferase, alkaline phosphatase, gamma-glutamyltransferase, creatine kinase and C-reactive protein. Significant changes were detected for fibrinogen, leukocytes and total protein. Increased leukocyte counts at baseline were likely caused by corticosteroid therapy before starting IA.

**Figure 2:**
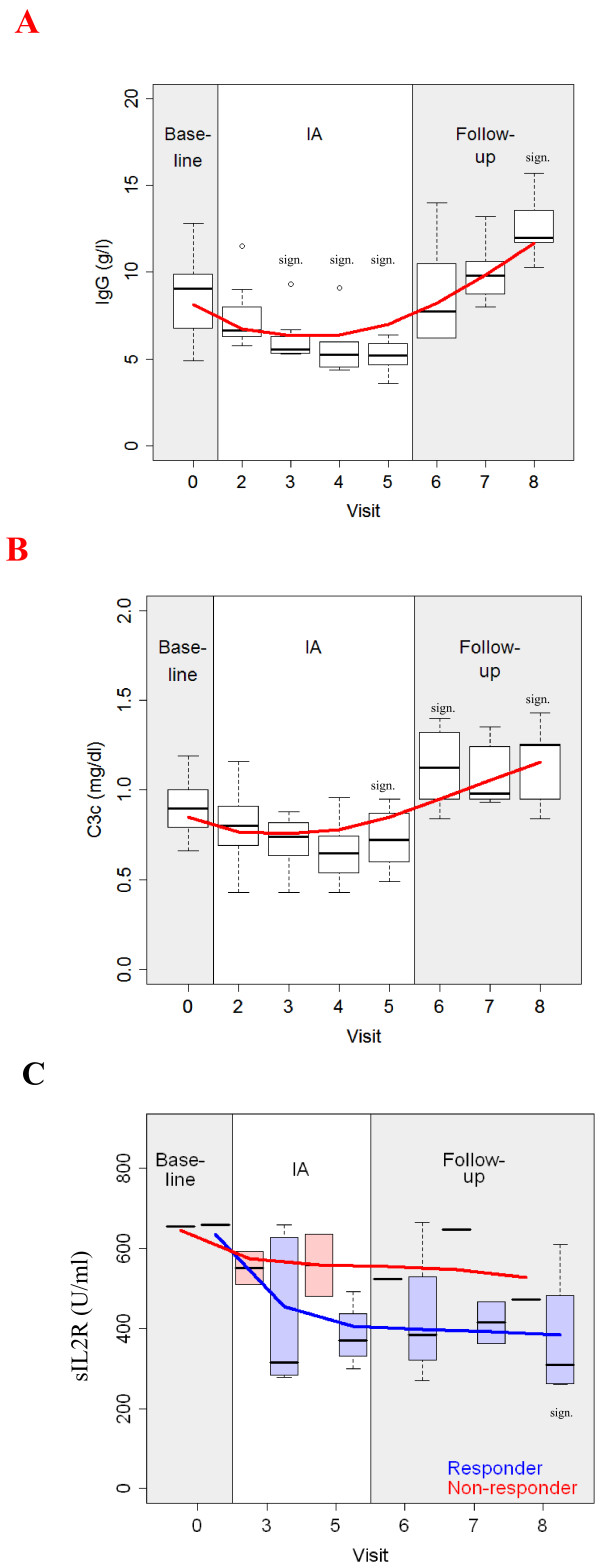
**Clinical chemistry.** Time-courses of (**A**) IgG; (**B**) C3c and (**C**) sIL-2R. sIL-2R is shown in responders (blue) and non-responders (red). Box plots: sign: significant (pairwise comparison to baseline).

Immunoglobulins IgA, IgG and IgM as well as complement components C3c and C4 significantly decreased during IA therapy (*P* < 0.01). After the end of IA, however, these parameters gradually recovered and increased beyond baseline values (as shown for IgG and C3c in Figure 2A, B). We found a significant decrease of sIL-2R at a rate of -35.4 ± 20.7% (*P* = 0.0054) after just one IA session in our patients with MS. Furthermore, time-courses of sIL-2R in responders and non-responders showed a significant difference (*P* = 0.03; Figure 2C). Sample dilution in the patients’ circulation due to IA was excluded by laboratory follow-ups of hematocrit and albumin in serum.

### **Proteomic data**

**Figure 3:**
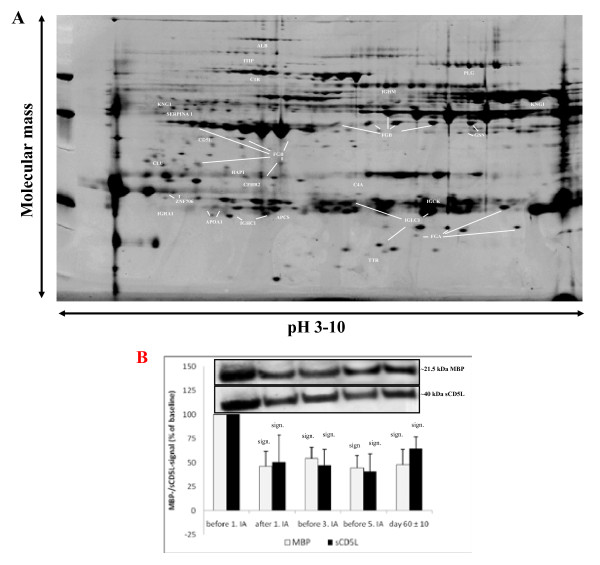
**Protein pattern of the eluate from the tryptophan column after the first immunoadsorption treatment in a single patient analyzed by two-dimensional gel electrophoresis.** (**A**) The protein spots were visualized by Flamingo fluorescence staining. (**B**) Time-course of plasma myelin basic protein fragment (molecular mass approximately 21 kDa) and soluble CD5L levels determined by western blot from a single patient with MS (upper panels) and densitometric analyses with baseline values set to 100% in the plasma of all 11 treated patients with MS before, during and after IA (lower panels). sign: significant (pairwise comparison to baseline).

Our proteomic investigation allowed the identification of 41 proteins with a peptide mass fingerprinting-score >65 in eluates of IA columns. We could identify apolipoproteins, hemorheologically relevant proteins, immunologically relevant proteins, myelin-related proteins and others. A representative two-dimensional gel is shown in Figure 3A and results are summarized in Table 3.

**Table 3:** List of identified proteins from immunoadsorption column elutes pooled from five patients with multiple sclerosis after the first immunoadsorption therapy

**Protein name**	**Gene name**	**UniProt**	**SwissProt**	**Nominal mass**	**PMF-score**
Alpha-1-antitrypsin	SERPINA1	A1AT HUMAN	57.5	46878	94
Apolipoprotein A-I	APOA1	APOA1 HUMAN	56.6	30759	152
Apolipoprotein A-IV	APOA4	APOA4 HUMAN	56.6	45371	67
CD5 antigen-like	CD5L	CD5L HUMAN	56.8	39603	120
Cleavage stimulation factor, subunit, tau variant	CSTF2T	CSTFT HUMAN	57.2	64624	57
Clusterin	CLU	CLUS HUMAN	56.8	53031	160
Complement C1r	C1R	C1R HUMAN	57.5	81606	72
Complement factor I	CF1	CFA1 HUMAN	56.8	68072	86
Complement factor H	CFH	CFAH	57.1	143680	61
Complement factor H-related protein 2	CFHR2	FHR2 HUMAN	57.4	30631	60
Complement C4-A	C4A	CO4A	57.4	192650	213
Fibrin alpha C term fragment		gi 223057	NCBI nr	14443	85
Fibrinogen alpha chain	FGA	FIBA HUMAN	57.1	95656	103
Fibrinogen beta chain	FGB	FIBB HUMAN	57.1	56577	170
Fibrinogen gamma chain	FGG	FIBG HUMAN	57.2	52106	146
Gelsolin	GSN	GELS HUMAN	57.2	86043	95
Histidine-rich glycoprotein	HRG	HRG HUMAN	57.0	60510	152
Huntingtin-associated protein 1	HAP1	HAP1 HUMAN	57.5	76208	65
Kininogen 1	KNG1	KNG1 HUMAN	56.8	72996	96
Transthyretin	TTR	TTHY HUMAN	56.6	15991	89
Immunoglobulin alpha-1 chain C region	IGHA1	IGHA1 HUMAN	57.4	37631	130
Immunoglobulin lamda chain C region	IGLC1	LAC HUMAN	57.4	11230	218
Immunoglobulin mu chain C region	IGHM	IGHM HUMAN	57.4	49960	90
Immunoglobulin kappa constant protein		gi 49258112		25915	78
Immunoglobulin kappa chain, C region	IGCK	IGCK HUMAN	57.2	11773	287
Immunoglobulin kappa light chain variable region		gi 48475436	NCBI nr	21251	80
Immunglobulin kappa light chain VLJ region		gi 21669479	NCBI nr	29086	76
Inter-alpha-trypsin inhibitor	ITIP	ITIH4 HUMAN	57.5	103521	67
Monoclonal immunoglobulin M antibody light chain		gi 41388186	NCBI nr	26008	65
Microtubule-actin crosslinking factor 1	MACF	MACF1	57.0	623626	57
Mps one binder kinase activator-like2A	MOBKL2A	MOL2A	57.0	25676	55
Myelin basic protein	MBP	MBP HUMAN	56.6	33097	55
Malate dehydrogenase cytoplasmic	MDH1	MDHC HUMAN	56.6	36631	57
Plasminogen	PLG	PLMN HUMAN	56.6	93247	89
Serum albumin	ALB	ALBU HUMAN	57.5	71317	101
Serum amyloid P-component	APCS	SAMP HUMAN	57.5	25485	68
Small ubiquitin-related modifier	SUMO1	SUMO1 HUMAN	57.2	11607	59
Transmembrane and tetratricopeptide repeat-containing protein 1	TMTC1	TMTC1 HUMAN	57.2	88209	62
Zinc finger protein basonuclin-2	BNC2	BNC2 HUMAN	57.2	1236677	62
Zinc fincer protein 706	ZNF706	ZN706 HUMAN	57.4	8606	64
39 S ribosomal protein L13 mitochondrial	MRPL13	RM13 HUMAN	57.0	20736	58

Gene names, accession number and identification score are given. PMF: peptide mass finger printing.

Amongst all identified proteins, soluble CD5L and the myelin basic protein (MBP) fragment were quantitatively analyzed in patients’ sera by western blots using specific antibodies (Figure 3B). The baseline value was set to 100% and follow-up values were given in percent of baseline. Plasma MBP levels significantly decreased during the first IA session to 46.4 ± 15.0% (*P* < 0.001) and remained low even in the post-IA control at day 60 ± 10. In parallel, soluble CD5L (sCD5L) levels decreased to 50.5 ± 15.0% (*P* < 0.001) after the first IA and remained subsequently low. Time-courses of sCD5L and MBP are shown in Figure 3B.

## **Discussion**

PE is an efficient treatment in acute central nervous system inflammatory demyelinating diseases [4], including severe optic neuritis, motor impairment or ataxia [5,6] after steroid-refractory relapses, successful in about 40% to 50% of cases. Here, we report on the first prospective investigation of tryptophan-IA in 11 relapsing MS patients with optic neuritis refractory to corticosteroid pulses in an open prospective study. Overall, eight of eleven patients (72.7%) achieved a remission. One patient gradually improved but deteriorated again along with the development of jugular venous thrombosis, and two patients did not respond at all. The response to IA seems to be comparable to the best results achieved in PE series [5,6] and to two very recent retrospective analyses of the effect of IA in steroid-refractory MS cases [18,19]. As in our own PE study [10], significant clinical improvement was seen after the third extracorporeal treatment session with a trend in favor of early IA initiation compared to delayed IA initiation. Our treatment protocol was limited to a total of five IA sessions. Additional experience showed that increasing the number of apheresis sessions did not correlate with further improvement of outcome (data not shown). This observation is in accord with results of IA in acute autoimmune neuropathies like Guillain-Barré Syndrome [20].

Most side effects were typical of any apheresis procedure using central venous lines as vascular access, but not characteristic of IA. Compared with the safety data from previously published PE studies [15,21], the incidence of mild adverse events was higher in our study, and moderate side effects were slightly more frequent. However, moderate side effects were almost level with our own study of neurological patients treated with PE [15]. Potential side effects of PE known to be related to the substitution of human plasma products were completely avoided [12].

Apart from clinical data, we analyzed possible therapeutic effects of IA with the help of proteomic analyses. Several relevant proteins, particularly fibrinogen and the immunoglobulins, were monitored. The decrease of fibrinogen is one limiting factor in the use of tryptophan-IA that makes regular controls necessary. Our protocol with five IA sessions on alternate days did not decrease fibrinogen to critical levels. Moreover, immunoglobulin depletion along with prior corticosteroid pulse therapy reflects a strong immunosuppression, which makes close controls of clinical and laboratory infection signs necessary. Previous investigations reported that the restoration of serum IgG levels until day 5 after IA does not result from increased antibody synthesis, but is probably related to changes of catabolism and immunoglobulin backflow [7]. Interestingly enough, we found a significant immunoglobulin increase beyond baseline values until day 180 ± 10 after the start of IA, suggesting additional mechanisms other than backflow alone.

Several mechanisms of PE action in neuroimmunological disorders have been described, such as a removal of pathogenic autoantibodies, a redistribution of pathogens from the extravascular to the intravascular compartment, increased proliferation of immune cells, an enhanced production of immunoglobulins, a promotion of suppressor T-cell function, and a deviation of cytokine patterns redressing a disturbed T-helper type 1 and T-helper type 2 balance [3]. Although IA has been termed specific, several studies have demonstrated additional binding properties of ligands other than immunoglobulins alone [22,23]. According to our proteomics data, several proteins that are possibly involved in MS pathogenesis are removed from the plasma by IA, for example, transthyretin [24], serum amyloid P [24], complement factors [24], clusterin [24], gelsolin [24], kininogen-1 [24], MBP [25,26], CD5L [27] and immunoglobulins [1,3,8]. We confirmed a decrease of serum levels by IA in two of them, MBP and sCD5L. MBP-like material has been detected in several body fluids including cerebral spinal fluid and the urine of patients with MS [28]. Since MBP and other myelin proteins have been shown to be encephalitogenic in animal models of MS, they could drive the systemic autoimmune response in patients with MS. Other investigations have demonstrated the prevalence of MBP-specific memory B-cells in the peripheral blood of relapsing patients with remitting MS that might prime T-cells in lymphoid organs to migrate into the central nervous system and to elicit IFN-γ secretion [25]. These data were further corroborated with the evidence of MBP-reactive T-cells among IL-2 expanded lymphocytes in patients with MS [26]. Thus, removal of MBP from the plasma by IA might interrupt these autoimmune mechanisms, although more research in this hypothesis is definitely needed.

sIL-2R, a marker of T_H_1 cell activation, is increased in the serum of patients with relapsing MS [14]. We induced a significant decrease of sIL-2R after IA in responders, but not in non-responders. Decreased sIL-2R levels might reflect the silencing of cellular autoimmune responses effective only in responders.

## **Conclusions**

Our clinical results show a high clinical efficacy of tryptophan-IA comparable to PE in the treatment of MS relapses refractory to corticosteroids. Furthermore, our experimental data suggest several possible effects on MS pathogenesis: not only removal of immunoglobulins and complement from plasma, but also reduced levels of circulating autoantigens and regulatory proteins. We suggest that more prospective studies are needed to confirm and extend our results, to give new insights into this treatment approach, to optimize therapeutic IA protocols and, lastly, to investigate further therapeutic principles, for example, T-cell reactivity to MBP before and after IA.

## **Abbreviations**

AE, Affected eye; CD5L, CD5 ligand; EDSS, Expanded Disability Status Scale; IA, Immunoadsorption; IFN, Interferon; Ig, Immunoglobulin; IL-2R, Interleukin-2 receptor; ISS, Incapacity Status Scale; kDa, kiloDalton; MBP, Myelin basic protein; MS, Multiple sclerosis; PBS, Phosphate-buffered saline; PE, Plasma exchange; PMF, Peptide mass fingerprinting; sIL-2R, Soluble interleukin-2 receptor.

## **Competing interests**

This work was supported by a research grant of Diamed (Cologne, Germany) to MK and BK. The authors declare lecture fees and/or travel funding from Diamed (Cologne, Germany) to MK, BK and GAM. MK received research funds from Novartis, Germany, and RK from Asahi Kasei Kuraray Medical, Japan.

## **Authors’ contributions**

MJK: design of the study, acquisition, analyses and interpretation of the data, drafting the manuscript, final approval. DT: acquisition and analyses of the data. MB: interpretation of the data, revising the manuscript. HD: analyses of the data. KJ: analyses of the data, revising the manuscript. DF: acquisition of the data, revising the manuscript. RK: design of the study, revising the manuscript. GAM: interpretation of the data, revising the manuscript. BK: design of the study, acquisition, analyses and interpretation of the data, drafting the manuscript, final approval. All authors read and approved the final manuscript.
